# Nutritional support for a person with type 1 diabetes undertaking endurance swimming

**DOI:** 10.3389/fendo.2022.1038294

**Published:** 2022-11-08

**Authors:** Siân Rilstone, Paul Spurway, Nick Oliver, Neil E. Hill

**Affiliations:** ^1^ Department of Nutrition & Dietetics, Imperial College Healthcare National Health Service (NHS) Trust, London, United Kingdom; ^2^ Department of Metabolism, Digestion and Reproduction, Imperial College London, London, United Kingdom; ^3^ Patient Representative, London, United Kingdom; ^4^ Department of Endocrinology & Diabetes, Imperial College Healthcare NHS Trust, London, United Kingdom

**Keywords:** carbohydrate, insulin, continuous glucose monitoring, open water swimming, protein

## Abstract

Long distance and open water swimming have increased in popularity over recent years. Swimming a long distance in lakes, rivers and the sea present numerous challenges, including cold water exposure and maintaining adequate nutritional intake to fuel exercising muscles. Guidelines exist outlining issues to consider and potential solutions to overcome the difficulties in feeding athletes. Exercising with type 1 diabetes adds further complexity, mostly around matching insulin to the recommended high carbohydrate intake, but also because of the way in which higher circulating insulin levels affect glucose utilisation and fat oxidation. This paper describes the nutritional considerations for people with type 1 diabetes intending to undertake long distance open water events, and insulin management suggestions to trial alongside. In addition, we include personal testimony from a swimmer with type 1 diabetes describing the challenges and considerations he faced when undertaking marathon swimming.

## Introduction

Around 400,000 people in the UK have type 1 diabetes (T1DM) (1). People with T1DM inject insulin to mimic physiological insulin secretion with basal insulin supplemented by boluses of rapid-acting insulin intended to match the absorption of glucose from ingested macronutrients. Insulin is administered either *via* subcutaneous injections or infusion using an insulin pump. Carbohydrate estimation with insulin dose adjustment improves glycaemic control (2) although can be challenging to undertake.

Glucose is the principal source of energy for exercising muscle and is available from skeletal muscle stores (in the form of glycogen) and from circulating glucose. Glucose entry to exercising muscle is limited by the GLUT4 transporter which is translocated to the cell membrane surface in response to activity, enabling targeted replacement of muscle glycogen stores as well as providing substrate for activity. GLUT4 transporters are additionally translocated to the cell membrane in response to insulin, potentiating glucose uptake in skeletal muscle (3). Ensuring a balance of available glucose and skeletal muscle uptake can be challenging with exogenous insulin administration, which also inhibits hepatic glucose release, and there is a significant risk of hypoglycaemia, both during and after exercise, in people with type 1 diabetes.

Adults with T1DM who exercise regularly tend to have a lower HbA1c, lower blood pressure, blood lipids and BMI than those who do not so should be encouraged to exercise, but may require help and support to do this safely (4, 5).

Marathon swimming is defined by FINA as a continuous swimming event of 10km distance, however the terms “marathon swimming” or “long-distance swimming” are used to refer to a range of distances, often, but not always, in open water (6). As with any outdoor long-distance event, open water swimming is challenging, with environmental factors frequently affecting events in an unpredictable way. In addition to humidity and solar radiation, open water swimmers must also account for tides and water temperature (7). Provision of adequate nutrition before, during and after a long-distance swim is therefore crucial and requires careful consideration. For example, hypothermia from cold water is a notable risk (8) and swimmers may try to accrue subcutaneous fat for insulation in preparation for a long-distance swim and use warm fluid feeds during an event with the aim of preventing core temperature from dropping (8). Open water swimmers often have high training volumes, focusing on high intensity, aerobic-dependent speeds (7).

People with type 1 diabetes who participate in endurance swimming events will have additional concerns, with many factors impacting health and performance. These concerns are noted from clinic consultation, personal accounts online (in forums such as runsweet.com). Published data are extremely limited in type 1 diabetes and endurance swimming, with some individual and small group case reports from triathlon competitions (9, 10). There will be overlap with those experienced in other endurance exercise events. Dietary changes, glycaemia and different insulin delivery systems have been explored in extreme events such as high-altitude mountain trekking (11, 12), a 1008km bicycle event (13) and ultramarathon running (14). The unique and overlapping factors impacting long-distance swimming will be explored in this paper, drawing on published papers, clinical guidelines, and the personal account of an endurance swimmer with type 1 diabetes.

## Nutritional considerations during exercise

Carbohydrate is the primary dietary source of glucose and thus is crucial to training. Insufficient carbohydrate intake can limit glycogen storage, which is associated with muscle fatigue during training (15). With high volume and intensity of training for open water swimming, athletes are encouraged to consume between 6-10g carbohydrate/kg/day (7). This increased carbohydrate intake may be challenging for a person with T1DM as they will require more insulin and will need to match the dose (in units) to the amount of carbohydrate consumed whilst considering the reduced insulin resistance associated with aerobic activity. Higher insulin doses can increase the risk of hypoglycaemia when exercise begins because exogenous insulin in a person with T1DM remains active at a time when people without diabetes would experience a drop in circulating insulin (4). Athletes with T1DM are encouraged to reduce their insulin doses before exercise to prevent hypoglycaemia (4), however care also needs to be taken to prevent a deficiency of insulin which would lead to hyperglycaemia and ketosis. A reduction in insulin dose may enable a switch to fat oxidation during exercise, which tends to be delayed in T1DM (because of the higher circulating insulin) leading to increased glucose disposal relative to hepatic glucose production (4). Strategies to adapt muscle to maximise the contribution of fat (obtained from adipose tissue) as an energy source should be used (16) to manage the long periods of submaximal work that marathon swimmers perform (17).

Periodized training involves a series of different strategies to improve the adaptive response to training and include training in the fasted state, with depleted storage glycogen, or withholding carbohydrate after training (16). These have not been studied in people with T1DM, in whom exercising without carbohydrate may make maintenance of target blood glucose levels difficult (4). Nevertheless, advice given to people with T1DM often recommends exercising in the fasted state with less active insulin ‘on board’ which may reduce blood glucose fluctuations.

### Carbohydrate intake during training

Glycogen stores are generally exhausted within 120 minutes of exercise depending on intensity and volume of stores (18) so exogenous supplies of carbohydrate are needed to sustain performance in endurance exercise. Benefits of consuming carbohydrate during training include:

- reducing the burden of carbohydrate consumption needed before and after training
- practicing feeding whilst training
- maximising performance by providing metabolic fuel (7)
- improving energy availability to prevent illness and injury. 30-60g carbohydrate per hour reduces counterregulatory hormone secretion during intense exercise, which can limit exercise-induced immunoparesis (19)
- allowing the athlete with diabetes to trial insulin adjustment plans with varying activity and carbohydrate

The amount of carbohydrate that can be absorbed and oxidised during exercise is related to the amount of carbohydrate the athlete habitually consumes. It is recommended that swimmers aim to practice consuming 90g carbohydrate/hour to maximise the carbohydrate utilisation during an event (7). A variety of carbohydrate sources should be recommended because the amount of glucose absorbed in the intestine is limited by the SGLT1 transporter (20). Different carbohydrates use different transporters, increasing the amount of carbohydrate that can be oxidised (21). A combination of glucose and fructose has been shown to raise blood glucose in a similar way to glucose alone (22). However, fructose can cause gastrointestinal discomfort, so should be trialled with caution (7).

### Nutritional management after exercise

To maximise replenishment of glycogen stores after endurance exercise, 1g carbohydrate/kg body weight should be consumed soon afterwards, with an ongoing carbohydrate intake of 1g/kg/hour for 4 hours after exercise. After that a normal eating pattern that provides all nutrients should continue, including a daily intake of 6-10g carbohydrate/kg/day (23).

People with diabetes are at risk of hypoglycaemia in the 24 hours after exercise due to the increased glucose disposal needed to replenish glycogen stores and increased insulin sensitivity. Basal insulin doses should be reduced by at least 20%, particularly following exercise in the afternoon or early evening, and bolus doses in the recovery phase often need reductions of 30-50% (4). Nausea, exhaustion, and satiety may occur, so carbohydrate that is easy to consume, such as in liquid form, should be considered. Glucose and, if necessary, ketone levels should be monitored closely. There may be additional benefit to consuming protein after exercise, to protect from late postprandial hypoglycaemia, as studies in non-exercising individuals with type 1 diabetes have shown that loads of 75g of protein or more resulted in glucose excursions 3-5 hours later (24).

Evidence suggests that high-medium glycaemic index (GI) foods result in greater glycogen storage after exercise than low GI carbohydrates (23). In people with T1DM, high GI carbohydrates can cause significant hyperglycaemia because of rapid absorption which may not be countered by exogenous insulin. It is important to discuss the timing of insulin to prevent a mismatch in circulating insulin and rapidly absorbed carbohydrate. A crossover study in people with T1DM found that giving an insulin bolus 20 minutes before a meal resulted in lower glycaemic excursions, lower blood glucose levels, and lower glucose area under the curve than the groups who gave their bolus at the start or 20 minutes after a standard meal (25).

Muscle protein synthesis is an essential element of training, recovery, and adaptation, with a role in tissue repair and the synthesis of sarcoplasmic and mitochondrial proteins in response to endurance exercise (23). The optimal protein dose after exercise is around 0.3g protein per kg body weight. This equates to 24g protein for a 80kg athlete, alongside 80g carbohydrate to provide the carbohydrate intake of 1g/kg/hour mentioned earlier. Ideally the rest of the protein for the day should be distributed at 3-5 hourly intervals at 4-5 opportunities and consumed in portions of 20-25g (26), ensuring that one of these is before bed (27). The type of protein may be as important as the amount - foods rich in protein containing all essential amino acids, such as milk, eggs, and meat, are associated with a significant increase in protein synthesis possibly related to quick digestion and a rapid rise in plasma leucine (23).

### Fat

Some athletes training for open water events in cold water will attempt to increase their subcutaneous fat for insulation (8). Consuming sufficient calories for a surplus to promote energy storage as fat can be very challenging when energy requirements for training are high. It is assumed that an increase in dietary fat, will increase subcutaneous fat, along with their visceral fat.

People with type 1 diabetes do not routinely give bolus insulin for dietary fat because it does not directly digest into glucose, and therefore is theoretically an attractive way to increase caloric intake. However, there is evidence to show that high fat meals (e.g., 60 g fat) can impact insulin requirements, possibly through an increase in insulin resistance (28). A high fat meal may, therefore, prevent the need for a reduction in the basal insulin overnight, which should be confirmed with regular glucose testing, and insulin dosing should be managed accordingly.

### Pre-event preparation

Carbohydrate loading involves consuming large amounts of carbohydrate (10-12g/kg body mass/day) for the 36-48 hours before an event (29) which results in supercompensation of glycogen stores i.e., storage of more glycogen than usual. Elevated starting muscle glycogen can postpone fatigue by approximately 20% in events lasting greater than 90 minutes (30). In endurance events such as a cross-Channel swim, these benefits may be less apparent, and there will be greater reliance on carbohydrate consumed during the event. High carbohydrate diets have been shown to improve performance in endurance events by just 2-3% (30), but, alongside adequate carbohydrate intake during the event, may be important, although their benefits should be weighed against the potential challenge of insulin dosing with this level of carbohydrate in athletes with type 1 diabetes.

A high carbohydrate breakfast on the morning of an event is recommended, containing a minimum of 1g/kg. Ideally this should be a low glycaemic index source of carbohydrate and should be low in fat (4). If this meal is 90-120 minutes prior to exercise, the mealtime bolus insulin should be reduced by 50-75% dependent on the expected intensity of the exercise to be undertaken (4).

The blood glucose level immediately prior to the event need to be monitored and may need require correction with additional carbohydrate if below 7-10mmol/l (31).

### Nutritional requirements during the event

The preservation of muscle glycogen during long distance swimming events (>5500m) is essential, as depleted glycogen stores have been shown to result in reduced distance per stroke, reduced stroke efficiency and higher energy expenditure (7). It is recommended that swimmers consume 90g carbohydrate per hour (32). When considering blood glucose management in an athlete with T1DM, 60g carbohydrate/hour is often recommended to stabilise glucose levels during activity (4). In this situation, where the athlete will have reduced their basal insulin by at least 20% and consumes 90g carbohydrate per hour, they may require small doses of rapid-acting insulin with feeds. The safest way to assess this is by monitoring glucose levels, and to trial insulin dosing and its effects on glucose levels during feeding in training.

The Channel Swimming and Piloting Federation (8) recommends finding a feeding pattern that suits the swimmer but points out that prolonged feeding adds significant time onto the overall duration of the cross-Channel attempt. They report the most common feeding strategy is to eat after the first hour, and then every half hour thereafter. It is recommended that feeds are kept to under 1 minute each. Feeding two or three times per hour may be more effective in people with type 1 diabetes (4) because it reduces the carbohydrate load and corresponding spikes in blood glucose (33).

Caffeine has demonstrated benefits in longer distance pool events and has been used in small doses along with carbohydrate at intervals in long distance events (7). Caffeine is commonly included in the final stages of a low carbohydrate training session, so may be useful towards the end of an endurance swim when glycogen stores may be depleted (34). The inclusion of caffeine should not cause concern in relation to its diuretic effect as the effect of dehydration and urine losses are minimal (23). Caffeine has been shown to limit reductions in blood glucose when 5-6mg/kg is given to athletes with T1DM before moderate to vigorous intensity aerobic exercise. Caffeine may be associated with late-hypoglycaemia, so it would not be recommended at the start of an endurance event such as channel swimming (35).

Caffeine is well-studied in physical activity of different durations and intensities. Its benefits are thought to be mostly due to stimulation of the central nervous system, however additional mechanisms are thought to play a role, including increased myofibrillar calcium availability and improvements in exercise metabolism and substrate availability (33).

Dietary nitrate, found in vegetables as NO3^-^, is converted into NO2^-^ then NO (nitric acid) in the body, and has been associated with vasodilation, blood flow and oxygen demand during exercise (36). The studies investigating the effect of nitrate have been of variable size and methodological design, with conflicting results. There are no published studies in athletes with type 1 diabetes, and therefore routine supplementation is difficult to justify. However, general dietary recommendations encourage a diet high in fruit and vegetables, so a diet rich in nitrate-containing vegetables such as spinach, beetroot and lettuce can be encouraged, and may result in a lower oxygen demand during suboptimal work (37).

Beta-alanine, which impacts intramuscular carnosine concentrations and is a popular supplement used in high intensity exercise, is yet to have shown a benefit in endurance exercise (38), nor swimming (39). Evidence to support supplementation in endurance swimming is therefore lacking, particularly in type 1 diabetes where no evidence currently exists at all.

Other supplements may be used as part of the preparation (such as creatine), but there are no data to suggest that they are safe or effective in type 1 diabetes

Fluid balance during long events in cold water must be carefully managed, as there is risk of hyponatremia in cold water swimming if fluid intake exceeds sweat losses. Therefore, concentrated carbohydrate sources can be used to meet the requirements for carbohydrate and energy without over hydration (7).

Assessing hydration status during aquatic sports is complex for many reasons (23), including difficultly in assessing losses, urine colour, and water intake from swimming. Requirements will depend on temperature, blood glucose levels, exertion, water and sodium intake from the open water. For this reason, training should include particular attention to fluid requirements and hydration status, to inform fluid intake during the event.

## Using technology to monitor blood glucose during exercise

Monitoring the effect of exercise and carbohydrate intake in an athlete with T1DM is crucial but can be challenging in water. Capillary blood glucose testing may be impossible as a capillary blood sample from the fingertip is required at a time when blood flow to the extremities will be reduced due to cold conditions and increased blood flow to the major muscle groups. Real-time continuous glucose monitoring (rt-CGM), in which the person with diabetes wears a sensor with a filament measuring the glucose concentration in the subcutaneous interstitial fluid, is a preferable option, as the sensor can be inserted prior to the activity, and glucose can be measured continuously, and displayed on a handset or mobile phone within 6 metres (i.e. on the support boat). This is a potential means of measuring glucose levels providing the sensor is worn somewhere it is not submerged in water, allowing transmission of the data in real-time to the support boat. Intermittently scanned CGM (isCGM) also known as flash glucose monitoring, using a device such as Freestyle Libre may be more practical since the device can be manually scanned by the support boat during feeds to obtain retrospective blood glucose data along with the current glucose level and trend. However, isCGM has been shown to be less accurate during acute exercise and hypoglycaemia (40) and its use is less likely to reduce rates of hypoglycaemia than with rtCGM outside of exercise (41).

Both rtCGM and isCGM rely on the glucose content of interstitial fluid in the tissue under the skin, which lags behind blood glucose by 5.3–6.2 minutes in healthy participants (42). This lag time has been shown to be influenced by many different factors including depth of sensor and hypoglycaemia (43)and has been shown to be significantly longer during physical activity, up to 35 minutes (44) but varies between different exercises and sensors (45, 46). In cold conditions, blood flow to the skin is reduced and so could potentially affect the accuracy of glucose monitoring.

The usability and safety of CGM is dependent on the accuracy of the sensors (47). This is often measured by mean or median absolute relative difference (ARD), commonly known as MARD, where sensor readings are compared to a reference blood glucose reading, ideally a venous sample measured on a laboratory analyser. Different sensors will have different MARDs in different settings, and there is debate as to what constitutes an appropriate MARD, with a MARD less that 10% often quoted as safe for providing information for calculating insulin doses (48). A review of the numerous studies testing MARD in exercise concluded that the overall mean of all MARDs during different types of exercise in people with type 1 diabetes is 13.63%, highlighting that the technology is not perfect. However, in endurance swimming it may be the only practical option for someone with type 1 diabetes to monitor glucose levels, and so should be used with an awareness that it could be over or under-estimating blood glucose levels (45, 49).

The Eversense continuous glucose monitor comprises an implanted optical glucose sensor component inserted in the subcutaneous space, a Bluetooth transmitter that attaches to the skin over the sensor and a smartphone receiver. The sensor is implanted for up to 180 days, is waterproof and has alarms for extremes of glucose, including a vibration alarm on the transmitter that may be useful for swimmers as it does not rely on a Bluetooth signal.

Automated insulin delivery systems have not been assessed in swimmers but may not operate as an end-to-end system underwater as they rely on a low-power Bluetooth signal, and may require a Smartphone.

Heart rate can provide some indication as to the intensity of the exercise, and has been used alongside oxygen uptake to estimate energy expenditure on a group level basis (50). This may be particularly important in type 1 diabetes, when percentage maximal heart rate can indicate whether the swimmer is likely to be using aerobic or anaerobic pathways, which will affect blood glucose levels.

Commencement of aerobic exercise, which is dependent on glucose, may need to be delayed if glucose levels are less than 7mmol/l, dependent on the direction of glucose travel, and during exercise glucose levels should be manipulated by carbohydrate consumption to ensure adequate glucose availability. Exercise should be suspended if glucose levels drop below 3.9mmol/l (31). Blood ketones levels can rise during prolonged exercise and can be influenced by low circulating levels of insulin, relatively high glucose levels, low carbohydrate diet and recent activity bouts. Blood ketone levels of 1.5mmol/l or more should be treated, and therefore exercise delayed (4).

## Insulin considerations in someone with type 1 diabetes

The absorption of subcutaneous insulin has been shown to be slower when there is more subcutaneous fat, and when the temperature of the skin is lower (51). During endurance exercise, a reduced level of circulating insulin is desirable (4), so these factors need to be considered when calculating reduced doses of insulin prior to the event. Conversely, at the end of the event when the swimmer is rewarmed, there is a risk of increased insulin absorption at a time when there is already a high risk of hypoglycaemia related to prolonged exertion (4). This can be mitigated by consuming carbohydrate immediately after exercise, which will also enhance recovery. Generally, people with type 1 diabetes are advised to halve their usual bolus insulin dose after exercise (4), but after an endurance event of this duration then a greater reduction may be required. Consideration should be given to the injection site and the corresponding speed of absorption (52) particularly regarding site and depth (53). Insulin pump therapy with a temporary basal rate feature is useful during exercise to reduce the level of circulating insulin, which may be particularly effective in prolonged exercise (54, 55). The practicalities of being connected to an insulin pump during exercise are individual, but many challenges have been solved with the introduction of patch pumps as an alternative to a tethered pump (56).

## Conclusion

The nutritional management of an endurance event is complex and includes consuming a diet that includes all the micronutrients as well as high intake of macronutrients, given in the right form at the right time. Ensuring adequate nutrition in an endurance open water event is challenging. When the athlete has T1DM, the issues that need to be considered are amplified, but with the right planning, practice and assessment the chances of success are optimised. Diabetes technology has an important role to play in maximising safety and success of endurance events.

## Personal testimony from a person with diabetes who is a long-distance swimmer

My interest in long distance swimming was borne from chronic knee problems that moved me on from running and cycling into the swimming pool. There are obvious challenges when swimming with diabetes that are exacerbated the longer you swim for, which increases the risk of hypoglycaemia and ketoacidosis. Symptoms of hypoglycaemia can be harder to identify when you are in the water and testing capillary blood glucose (CBG) levels can be very challenging. CBG testing is essential to identify hypo and hyperglycaemia, and the effect that exercise has on your glucose levels, as well as the effect of the nutrition required to undertake a marathon swim. An understanding of all these factors is essential to give you confidence entering a marathon swimming event.

Before engaging with the team at Imperial College Healthcare NHS Trust I had a very crude mechanism for evaluating my blood glucose responses which included only testing before and after the swims. Ensuring there was enough glucose in my body to perform the exercise was achieved by loading with carbohydrate before the swim. If the goal is to then swim for marathon swims in excess of 10km and durations upwards of 3 hours, feeding every 30-40 minutes, then there is a real need to understand how your body responds to the food and the exercise.

To get an understanding of what happens between feeds on these longer swims regular testing is the obvious solution which is valid and feasible when swimming in a pool. It does however interrupt your training and the devices are potentially exposed to water damage when sitting next to the pool. In open water swimming even with a support boat or kayak it becomes very challenging to get a glucose reading with all the water around.

I used an Abbott Freestyle Libre device which was scanned at feeding time. The glucose data can be displayed on the receiver held by a person on the support boat. I wore the sensor on the fatty tissue on or near the triceps but raising the arm at feeding time for a reading was sometimes challenging.

I have experience of several long swimming events and have learnt a great deal about my glucose response in the process. I have trialled different long-acting insulin doses on the morning of the event, recognising that I need some background insulin, but not so much that it will cause hypoglycaemia when I start exercising, as I swim most days, I find that this dose is the same whether I have an event or training.

When I did the Torbay 8 mile open water swim my routine was as follows:

6.30am: 13 units Glargine

8 units Novorapid with muesli and fruit (aiming for food to have digested and insulin have finished acting by the start of the race)

Snacks of nuts and fruit between breakfast and swim start

10.45am: Hot gel shot (21g carbs) immediately before the official swim start.

Feed during swim (every 40 minutes):

Feed 1: 200ml mix of warm lime shot with Gatorade (32g carbohydrate)
Feed 2: 200ml mix of warm caffeine gel with Gatorade (32g carbohydrate)
Feed 3: 200ml warm ovaltine (15g carbohydrate)
Feed 4: 200ml mix of warm lime shot with Gatorade (32g carbohydrate)
Feed 5: 100ml warm coke (11g carbohydrate)
Feed 6: 100ml warm coke (11g carbohydrate)

**Figure 1:**
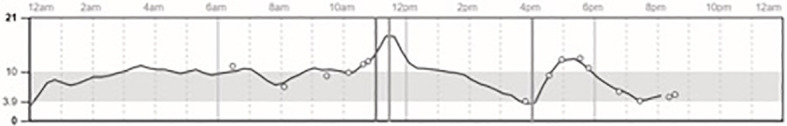
Libre trace during the Torbay 8 mile swim.

The graph below shows my glucose trace from my libre sensor (**Figure 1**):

My learning points:

-The high blood glucose at the start of the event was unplanned but explainable as the event started late - 20 mins after the gel which was intended to be taken immediately before the race.
-The low glucose level at the end of the swim highlighted how important it is to take the full feed no matter how full you are or how close you think you are to the finish – a very interesting learning point for me.

**Figure 2:**
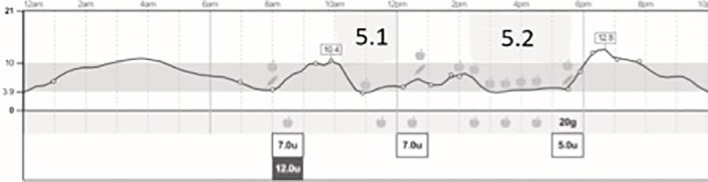
Glucose trace from swim camp.

Shortly after the 8-mile swim, I attended a Swim Camp. One of the days glucose profile is shown below (**Figure 2**):

This swim highlighted to me how swimming can be both aerobic and anaerobic depending on intensity, and these two situations need to be managed differently. Continuous glucose monitoring has been essential to enable me how to understand how exercise, insulin and carbohydrate affect my glucose levels, and to provide data the data for my support team during events to anticipate problematic situations and respond accordingly.

## Author contributions

SR wrote the review part of the manuscript and advised PS. PS wrote the first-person account part of the manuscript, undertook swimming training and events. NH advised PS and contributed to the manuscript. NO contributed to the manuscript and advised on CGM.

## Acknowledgments

This paper was written following a consultation in Imperial College Healthcare NHS Trust Physical Activity and Diabetes clinic.

## Conflict of interest

Author NO has received honoraria for advisory board participation and for speaking from Roche Diabetes and Dexcom, and has received research funding from Dexcom and Roche Diabetes. Author NH has received continuous glucose monitoring equipment and study funding from Dexcom San Diego, California for research.

The remaining authors declare that the research was conducted in the absence of any commercial or financial relationships that could be construed as a potential conflict of interest.

## Publisher’s note

All claims expressed in this article are solely those of the authors and do not necessarily represent those of their affiliated organizations, or those of the publisher, the editors and the reviewers. Any product that may be evaluated in this article, or claim that may be made by its manufacturer, is not guaranteed or endorsed by the publisher.
